# Measuring symptoms of obsessive-compulsive and related disorders using a single dimensional self-report scale

**DOI:** 10.3389/fpsyt.2023.958015

**Published:** 2023-02-14

**Authors:** Beatriz Moreno-Amador, José A. Piqueras, Tíscar Rodríguez-Jiménez, Agustín E. Martínez-González, Matti Cervin

**Affiliations:** ^1^Health Psychology Department, Miguel Hernández University, Elche, Spain; ^2^Department of Psychology and Sociology, University of Zaragoza, Teruel, Spain; ^3^Department of Developmental Psychology and Didactics, University of Alicante, Alicante, Spain; ^4^Department of Clinical Sciences Lund, Lund University, Lund, Sweden; ^5^Child and Adolescent Psychiatry, Lund, Sweden

**Keywords:** obsessive-compulsive (OC) spectrum disorders, obsessive-compulsive and related disorders, body dysmorphic disorder, hoarding disorder, hair-pulling disorder, skin-picking disorder, adolescents, adults

## Abstract

**Background:**

Obsessions and compulsions are heterogenous but can be classified into obsessive-compulsive disorder (OCD), body dysmorphic disorder (BDD), hoarding disorder (HD), hair-pulling disorder (HPD), and skin-picking disorder (SPD). OCD is in itself heterogenous, with symptoms clustering around four major symptom dimensions: contamination/cleaning, symmetry/ordering, taboo obsessions, and harm/checking. No single self-report scale captures the full heterogeneity of OCD and related disorders, limiting assessment in clinical practice and research on nosological relations among the disorders.

**Methods:**

To provide a single self-report scale of OCD and related disorders that respects the heterogeneity of OCD, we expanded the DSM-5-based Obsessive-Compulsive and Related Disorders-Dimensional Scales (OCRD-D) so that is also includes the four major symptom dimensions of OCD. A psychometric evaluation and an exploration of the overarching relations among the dimensions were conducted using an online survey which was completed by 1,454 Spanish adolescents and adults (age span = 15–74 years). Approximately 8 months after the initial survey, 416 participants completed the scale again.

**Results:**

The expanded scale showed excellent internal psychometric properties, adequate test-retest correlations, known groups validity, and correlations in the expected directions with well-being, depression/anxiety symptoms, and satisfaction with life. The higher-order structure of the measure indicated that harm/checking and taboo obsessions formed a common disturbing thoughts factor and that HPD and SPD formed a common body-focused repetitive behaviors factor.

**Conclusion:**

The expanded OCRD-D (OCRD-D-E) shows promise as a unified way to assess symptoms across the major symptom dimensions of OCD and related disorders. The measure may be useful in clinical practice (e.g., screening) and research, but more research on construct validity, incremental validity, and clinical utility is needed.

## Introduction

Intrusive thoughts and compulsive behaviors are common in humans, and when time-consuming and/or impairing, they can constitute a mental disorder. Among disorders revolving around obsessions and compulsions, obsessive-compulsive disorder (OCD) has attracted most research attention. OCD is characterized by obsessions (repetitive and persistent thoughts, urges, or images) and compulsions (repetitive behaviors or rituals performed to alleviate obsessions-related distress or prevent harm) (1). OCD affects 1–2% of the population (2, 3), but several other disorders related to obsessions and compulsions have been suggested. Accordingly, in the fifth edition of the Diagnostic and Statistical Manual of Mental Disorders (DSM-5), body dysmorphic disorder (BDD), hoarding disorder (HD), hair-pulling/trichotillomania disorder (HPD), and skin-picking/excoriation disorder (SPD) were included alongside OCD in a new chapter called Obsessive-Compulsive and Related Disorders (OCRD) (1).

The major characteristics of BDD are preoccupations with perceived appearance flaws and associated repetitive behaviors. HD is characterized by a persistent difficulty to discard or part from possessions because of a perceived need to save or distress associated with discarding items. HPD and SPD are characterized by hair-pulling and skin-picking behaviors, which are not performed to harm oneself or for esthetical reasons. Individuals with OCD, BDD, HD, HPD, and SPD have reduced or no control over symptoms, commonly avoid places or people because of the symptoms, and experience distress or impairment (1).

The disorders included in the OCRD chapter in DSM-5 were grouped because they revolve around repetitive behaviors and share key etiological factors, such as genetic risk (4, 5), and family history (6). The new chapter was also introduced to stimulate research on the related disorders and ultimately to improve assessment and treatment (7), and at least the former has been achieved (8). Nevertheless, the new chapter has generated controversy and there is no consensus on the optimal nosological placement of the OCRD disorders and whether they are more closely related to each other than to other psychiatric disorders (e.g., anxiety disorders) (9, 10). Specifically, the inclusion of HPD and SPD alongside OCD is debated, with some authors arguing that HPD and SPD are not clearly related to OCD and BDD, which are suggested to be closely related to each other (and to anxiety disorders) (11). Some evidence in favor of this hypothesis was recently provided by a cluster-analytic study showing that individuals with elevated OCD and BDD symptoms belonged to a “compulsive” or “mixed” cluster and those with HD symptoms to an “impulsive” group, while those with HPD and SPD symptoms did not fit any cluster (12).

The diagnostic description of OCD also changed in the DSM-5, as compulsive hoarding was excluded from OCD and placed under its own diagnostic category. The exclusion of hoarding from OCD relied on empirical studies showing that OCD is highly heterogenous, with symptoms revolving around thematically coherent symptom dimensions. The OCD symptom dimension model with the most empirical support includes four dimensions: (1) *symmetry*: symmetry obsessions and repeating, ordering, and counting compulsions; (2) *forbidden thoughts*: aggression, sexual, religious, and somatic obsessions and checking compulsions; (3) *cleaning*: cleaning and contamination; and (4) *hoarding*: hoarding obsessions and compulsions. This four-factor structure typically explains a large proportion of the heterogeneity in the clinical presentation of OCD symptoms (13). Recent work, based on a more comprehensive pool of OCD symptoms, replicated the four dimensions but suggested four additional dimensions (superstition, transformation concerns, body-focused symptoms, and loss/separation) (14).

The symptom dimensions of OCD have been shown to be underpinned by differences in etiology and brain correlates (15), making them important to consider in research on OCD. Research has also showed that while specific symptoms may fluctuate and vary, individuals with OCD generally stay within their major symptom dimension over time (16), indicating that the symptom dimensions of OCD have prognostic value. Relations between the OCD symptom dimensions and the related disorders in the OCRD chapter is scarce (17), and it is unclear whether some OCD dimensions are more strongly related to BDD, HD, SPD, and HPD. Clarity on this issue is important for research on etiology, nosology, and classification of OCD and related disorders.

The heterogeneity of OCD can be examined using several different measures. Nevertheless, the only measure that respects the empirically supported symptom dimension model of OCD is the Dimensional Yale Brown Obsessive Compulsive Scale (DY-BOCS) (18), which includes the dimensions identified in the meta-analysis by Bloch et al. (13). However, DY-BOCS does not include sections for BDD, HD, HPD, and SPD. For the latter, there are specific measures, such as the Yale-Brown Obsessive-Compulsive Scale Modified for BDD (BDD-YBOCS) (19), the Hoarding Rating Scale-Self Report (HRS-SR) (20), the Massachusetts General Hospital Hair Pulling Scale (MGH-HPS) (21, 22); and the Skin Picking Scale-Revised (SPS-R) (23).

To aid research, the DSM-5 Obsessive-Compulsive and Related Disorders workgroup developed a self-report scale that can be used to assess BDD, HD, HPD, and SPD using a single scale. The measure is called the Obsessive-Compulsive and Related Disorders-Dimensional scales (OCRD-D) (24) and is based on the Florida OCD Inventory (FOCI), developed by Storch et al. (25), which has demonstrated strong psychometric properties, including a strong correlation with the clinician-rated Y–BOCS (25). The FOCI consists of two modules. First, the individual responds “yes” or “no” to 20 cognitive, affective, and behavioral symptoms of OCD. Second, five broad severity questions (time, distress, impairment, avoidance, and control), which largely correspond to the severity items of Y-BOCS (but resistance in Y-BOCS has been replaced by avoidance) are completed. The OCRD-D is based on the second section of the FOCI and uses the five severity items to measure BDD, HD, HPD, and SPD symptoms. The five items assess: (1) time occupied by the symptoms (for HD: difficulty discarding [or recycling, selling, giving away] ordinary things that other people would get rid of), (2) distress caused by the symptoms, (3) difficulty controlling the symptoms (for HD: how difficult is to use the rooms in the home because of the clutter or number of possessions), (4) avoidance because of the symptoms, and (5) interference in school, work, social, and family life due to the symptoms. These items broadly reflect (although not precisely) the proposed diagnostic criteria for OCRDs in DSM-5 and are completed after a vignette describing the core features of each disorder.

The OCRD-D makes it possible to simultaneously assess symptoms of BDD, HD, SPD, and HPD. This can greatly benefit research as it reduces problems related to measurement artifacts (e.g., that measures using similar response scales become more strongly associated than measures using different response scales). Further, most current self-report measures of OCD and related disorders rely on frequency ratings of specific symptoms (e.g., I wash my hands repeatedly) and not overall symptom severity. In contrast, the OCRD-D uses a broad thematic description of the core symptoms of each disorder and then five broad items (i.e., not related to only specific symptoms) to assess overall severity. The thematic presentation of symptom dimensions in the OCRD-D makes it preferable to the FOCI, where the presence of OCD symptoms is assessed using 20 specific symptom items. Thus, the OCRD-D is both more time efficient and less reliant on the presence of specific symptoms than most other current measures of OCD and related disorders. A limitation with the OCRD-D is that OCD is not included. Further, given that the heterogeneity of OCD may be linked to differences in etiology, it would be preferable to include modules designed to measure at least the four major OCD dimensions of the DY-BOCS, that is, symmetry/ordering, aggressive symptoms (e.g., harm/checking), taboo thoughts, and contamination/cleaning. A measure that includes the major symptom dimensions of OCD as well as BDD, HD, SPD, and HPD symptoms would greatly benefit research on OCRD disorders, for example, how they relate to each other.

The main objectives of this study are to (i) expand the OCRD-D to include the major symptom dimensions of OCD, (ii) examine the psychometric properties (factor structure, internal consistency, construct validity, test-retest reliability) of the expanded measure in a community-based sample of Spanish adolescents and adults, and (iii) explore the overarching factor structure of the measure to provide clarity on how the disorders in the OCRD chapter are related while appreciating the heterogeneity of OCD.

## Materials and methods

### Participants and procedure

The project from which data for this study are drawn includes three online surveys completed over a period of 18 months. Here we analyze data from the first (T0) and second (T1) surveys; the third (T2) survey is ongoing. The sample consisted of 1,454 (T0) and 416 (T1) Spanish adolescents and adults aged 14–64 years (T0: M = 23.84, *SD* = 8.46; and T1: M = 26.21, *SD* = 7.47). Participants were recruited using online advertisement, advertisement through organizations associated with mental health, especially those pertaining to OCD and related disorders, and through secondary education centers. Of the full sample, 163 participants (11.2%) reported that they had received an OCD diagnosis at some time in their life and 135 participants (9.3%) reported that they had received a diagnosis of either HPD or SPD. The strategy to oversample individuals with higher levels of symptoms aligns with the recommendation of the Research Domain Criteria (RDoC) initiative when studying psychopathology using a dimensional approach (26). The first survey was conducted between October 2020 and October 2021 and the second survey between June 2021 and December 2021, with a difference between T0 and T1 of around 8 months. Participants completed study measures online after having read and accepted an informed consent. The survey was conducted using the online survey tool Lime Survey© and took approximately 30 min to complete. Participation was voluntary, and participants had the chance to win gift cards with a value of $100. Table 1 presents sociodemographic information of the T0 and T1 samples.

**TABLE 1:** Sociodemographic characteristics of the sample.

Variables	T0	T1
Age, M (SD)	23.84 (8.46)	26.21 (7.47)
14–18 years old, *n* (%)	469 (32.3%)	21 (5%)
19–25 years old, *n* (%)	580 (39.9%)	225 (54.1%)
26–30 years old, *n* (%)	194 (13.3%)	92 (22.1%)
>30 years old, *n* (%)	211 (14.5%)	78 (18.8%)
Total	1454 (100%)	416 (100%)
**Sex**		
Females, *n* (%)	1041 (71.5%)	319 (76.5%)
Males, *n* (%)	404 (27.6%)	96 (23.0%)
Other, *n* (%)	1 (0.1%)	0 (0.0%)
Not want to answer	8 (0.6%)	2 (0.5%)
**Gender**		
Females, *n* (%)	1028 (71.5%)	314 (76.6%)
Males, *n* (%)	397 (27.6%)	94 (22.9%)
Other, *n* (%)	3 (0.2%)	0 (0.0%)
Not want to answer	10 (0.7%)	2 (0.5%)
**Country/Region of birth**		
Spain, *n* (%)	1341 (92.2%)	410 (98.6%)
Rest of Europe, *n* (%)	32 (2.2%)	5 (1.2%)
America, *n* (%)	66 (4.5%)	0 (0%)
Africa, *n* (%)	10 (0.7%)	1 (0.2%)
Asia, *n* (%)	5 (0.3%)	0 (0%)
**Country/Region of residence**		
Spain, *n* (%)	1438 (98.9%)	412 (99%)
Rest of Europe, *n* (%)	14 (1.0%)	3 (0.7%)
Asia, *n* (%)	1 (0.1%)	1 (0.2%)
**Spanish participants**		
Origin and residence in Spain	1325 (91.1%)	409 (98.3%)
Origin or residence in Spain	129 (8.9%)	17 (1.7%)
**Education level**		
Secondary, *n* (%)	448 (36.9%)	117 (36.1%)
Superior, *n* (%)	766 (63.1%)	207 (63.9%)
**Presence of psychological problems**		
Body focused repetitive behaviors, *n* (%)	135 (9.3%)	52 (12.5%)
Obsessions, *n* (%)	147 (10.1%)	51 (12.3%)
Compulsions, *n* (%)	86 (5.9%)	28 (6.7%)

### Measures

#### The obsessive-compulsive and related disorders dimensional scales-expanded version (OCRD-D-E)

We expanded the OCRD-D developed by LeBeau et al. (24). Several studies have shown that the original measure and its domains/modules have good psychometric properties (24, 27–29). Further, OCRD-D has been validated in Spanish adolescents and exhibited good psychometric properties (30). We expanded the measure by including the four major symptom dimensions of OCD: contamination/cleaning, harm/checking, symmetry/ordering, and taboo obsessions. We used descriptions of each symptom dimension similar to those used in the DY-BOCS (18) and the Dimensional Obsessive-Compulsive Scale (DOCS) (31) and added elements mentioning common emotional themes in each dimension (32, 33). Similar descriptions have been used to successfully screen for the major symptom dimensions of OCD in children and adolescents as part of an interview-only version of the DY-BOCS (34). Further, the description of BDD, HD, HPD, and SPD were expanded to better capture the full phenomenology of symptoms within each module. The expanded measure (in both Spanish and English) can be found in the Supplementary material.

In the expanded OCRD-D (hereafter: OCRD-D-E), the severity of each OCD symptom dimension was scored using the same five severity items as for the other OCRD-D scales (time, distress, control, avoidance, and interference; for more information, see Section “Introduction” and the full scale in the Supplementary material). All items are rated on a 5-point Likert-type scale yielding a total score of 0–20 for each module, with higher scores indicating greater severity. For items 1, 2, 4, and 5, the anchors range from “None” to “Extreme.” For item 3, the anchors range from “Complete control” to “No control.”

To buffer against daily/weekly fluctuations, symptom severity during the last month was reported instead of symptom severity during the last 7 days, as in the original version. This change was made to provide information on the frequency of symptoms in terms of current prevalence, with “past month” being the most common period used to assess for OCD (35). Furthermore, the original measure, which uses “the last 7 days,” was developed to be administered at an initial patient interview and to monitor treatment progress (1). In this sense, Shrout et al. (36) determine that including a shorter time frame in a measure increases its sensitivity to change. Because the present study uses a community sample and not clinical participants, we considered that the “last 7 days” might lead to false negatives and not discriminate the presence of current symptomatology. Similarly, Streiner et al. (37) concluded that “a 1-month period is an appropriate compromise between the need for a window of some duration to allow for the fact that most disorders do not have an acute onset, with a measure that is responsive to changes in health care delivery” (p. 226).

In addition to changing the time frame, we made some minor additions to the original measure. In the HPD and SPD modules, when asking about hair-pulling or skin-picking behaviors, we added the caveat “out of habit, not with the purpose of harming yourself.” This was done to avoid assessing skin picking or hair pulling that occurs in the context of non-suicidal self-injury (1). We deleted “skin picking” from examples of repetitive behaviors in the BDD module to minimize artificial overlap between BDD and SPD. Finally, in the OCD modules, we provided examples of typical OCD symptoms (e.g., fear of contamination and checking compulsions) to avoid assessing repetitive thoughts and behaviors related to other conditions (e.g., worry or rumination). Further, for the OCD dimensions of contamination/cleaning, we added the caveat that “when answering this question, think about your thoughts/behaviors in general, and not specifically those due to the current exceptional situation regarding the COVID-19 pandemic, where these thoughts and behaviors of hygiene and social distance are sanitary measures assumed by the whole society.”

The OCRD-D-E was applied as a self-report measure and completion of each dimension/module took between 20 s and 3 min across participants; the total response time (8 modules) ranged from 2.5 to 24 min.

### External validators

#### The 5-item World Health Organization well-being index (WHO-5)

The WHO-5 (38) is a brief scale measuring subjective well-being in terms of positive mood, vitality, and general interest, with 5 items rated on a 6-point Likert scale (0–5), from “Never” to “All the time.” The scale is widely used and has adequate validity as a screening tool for depression (39). In the present study, the items of the measure showed good internal consistency (α = 0.90). The measure was used to examine convergent validity, and we expected that all domains of the OCRD-D-E would be negatively associated with well-being.

#### The brief patient health questionnaire (PHQ-4)

The PHQ-4 (40) is a brief self-report screening measure for depression and anxiety with 4 items, two per each factor, with each item being rated on a 4-point Likert scale (0–3), from “Never” to “Almost every day.” Its validity and reliability are satisfactory in the general population (41) and in the present study, the measure showed good internal consistency for depression (α = 0.81), anxiety (α = 0.84), and the total score (α = 0.85). The PHQ-4 was used to examine convergent validity, and we expected that all domains of the OCRD-D-E would be positively associated with depressive/anxiety symptoms.

#### Satisfaction with life

To assess life satisfaction, we used a single 11-point Likert item ranging from 0 (no satisfaction) to 10 (extreme satisfaction). This one-item measure was used since previous research has shown that single-item measures of life satisfaction have similar convergent validity as multiitem scales [e.g., (42, 43)]. The measure was used to examine convergent validity, and we expected that all domains of the OCRD-D-E would be negatively associated with satisfaction with life.

### Data analyses

To psychometrically evaluate the OCRD-D-E, we used confirmatory factor analysis (CFA). Diagonally weighted least squares estimation was used because the items of the OCRD-D-E were ordinal. Model/data fit was evaluated using four scaled fit indexes: CFI and TLI (values > 0.95 are indicative of good fit and values > 0.90 of adequate fit) and RMSEA and SRMR (values < 0.06 and 0.08, respectively, are indicative of good fit) (44). The primary theoretical model was a model with eight first-order factors (the four OCD dimensions plus HD, BDD, HPD, and SPD). This model was contrasted with a unidimensional model where a single factor explained correlations among items. Complete data for all items were available. Internal consistency of the items of each domain was computed using Cronbach’s alpha (α) and McDonald’s omega (ω). All CFA and internal consistency analyses were conducted using the R libraries *lavaan* (45) and *semTools* (46). To estimate the association between the OCRD-D-E domains and external validators (satisfaction with life, anxiety/depression [PHQ], and well-being [WHO-5]), we used the R library *latentcor* (47) that implements methods that can estimate correlation coefficients among zero-inflated variables and for different types of variables (e.g., continuous, ordinal, zero-inflated) using the latent Gaussian copula model (48). Known groups validity was examined by comparing scores on the eight modules of the OCRD-D for those with/without a lifetime history of an OCD diagnosis and HPS/SPD diagnosis, respectively. Independent samples Mann–Whitney *U* tests were used because of the non-normal properties of the data, but Cohen’s *d* was used to indicate the size of each difference. Known groups validity was examined using SPSS, version 27. Test-retest reliability was examined using Pearson correlations between the T0 and T1 data for each factor in R.

To explore the overarching factor structure of the symptom dimensions, we split the sample into two random halves and conducted exploratory factor analysis (EFA) on the eight OCRD-D-E domains (sum scores) using the first half. To estimate the correlation matrix of the variables, we again used the R library *latentcor* and estimated correlations using the latent Gaussian copula model. We then estimated the overall Kaiser–Meyer–Olkin (KMO) Test value and the Bartlett’s test of sphericity to explore whether the data were suitable for EFA. Parallel analysis was used to explore the number of factors to retain. Then, principal axis factoring and promax rotation was used to extract the factors. The empirically derived factor structure was then tested using CFA in the other half of the sample and model/data fit evaluated.

## Results

### Psychometric properties of the OCRD-D-E

The model/data fit of the proposed factor structure with eight first-order factors (that were allowed to correlate freely) was excellent (CFI = 0.99, TLI = 0.99, RMSEA = 0.04, SRMR = 0.04). In contrast, the unidimensional factor structure fitted the data very poorly (CFI = 0.80, TLI = 0.79, RMSEA = 0.17, SRMR = 0.33). In the factor structure with eight first-order factors, all items loaded statistically significantly onto their proposed factor (*p*s < 0.001) and all items had standardized loadings above 0.80, except one item from the HD-D scale: “Because of the clutter or number of possessions, how difficult is it for you to use the rooms in your home” (standardized loading = 0.78). The internal consistency of each of the eight first-order factors was excellent: harm/checking (α = 0.90, ω = 0.92, average item variance explained [AVE] = 79%), taboo obsessions (α = 0.92, ω = 0.94, AVE = 87%), symmetry/ordering (α = 0.91, ω = 0.93, AVE = 82%), contamination/cleaning (α = 0.95, ω = 0.96, AVE = 90%), BDD (α = 0.95, ω = 0.96, AVE = 89%), HD (α = 0.88, ω = 0.91, AVE = 77%), HPD (α = 0.95, ω = 0.97, AVE = 96%), and SPD (α = 0.90, ω = 0.95, AVE = 96%). The test-retest reliability of each scale was 0.56 for harm/checking, 0.60 for taboo obsessions, 0.67 for symmetry/ordering, 0.63 for contamination/cleaning, 0.63 for BDD, 0.56 for HD, 0.79 for HPD, and 0.62 for SPD. All test-retest correlations were statistically significant at the 0.01 level.

### Construct validity

The associations between the dimensions of OCRD-D-E and well-being, depression/anxiety, and satisfaction with life are presented in Table 2. All correlations were estimated using the latent Gaussian copula model and were statistically significant (*p* < 0.001) and in the expected direction. Known groups validity was examined by comparing scores on each of the eight OCRD-D-E domains for those with/without a lifetime history of an OCD diagnosis or an HPD/SPD diagnosis, respectively, using Mann–Whitney *U* tests. Results are presented in Table 3 and supported construct validity since those with a lifetime history of OCD scored significantly higher on all variables, with the largest differences emerging for the OCD variables, while those with a lifetime history of HPD/SPD scored significantly higher on 6 of the 8 variables, with the largest differences emerging for the HPD and SPD variables.

**TABLE 2:** Means and standard deviations for all OCRD-D-E scales and correlations with satisfaction with life, depression/anxiety symptoms, and well-being.

OCRD symptom dimensions	*M* (*SD*)	Satisfaction with life	Depression and anxiety	Well-being
Harm/Checking	3.25 (4.13)	−0.33	0.47	−0.36
Taboo obsessions	1.75 (3.57)	−0.22	0.27	−0.17
Symmetry/Ordering	3.93 (4.47)	−0.23	0.37	−0.26
Contamination/Cleaning	2.74 (4.42)	−0.10	0.26	−0.15
Body dysmorphic	6.63 (5.89)	−0.41	0.47	−0.37
Hoarding	2.56 (3.58)	−0.22	0.33	−0.30
Hair-pulling	1.43 (3.93)	−0.25	0.33	−0.30
Skin-picking	2.70 (4.13)	−0.23	0.30	−0.25

All correlations are significant at the *p* < 0.01 level and estimated using the latent Gaussian copula model. OCRD-D-E, obsessive-compulsive and related disorders-dimensional scales-expanded version.

**TABLE 3:** Differences on the OCRD-D-E for those with vs. without a lifetime history of an OCD diagnosis and those with vs. without a lifetime history of an HPD/SPD diagnosis.

	OCD, *M* (*SD*)	No OCD, *M* (*SD*)	*P* for difference	Cohen’s *d*
Harm/Checking	7.99 (5.79)	2.66 (3.44)	<0.001	1.41
Taboo obsessions	5.06 (6.27)	1.33 (2.80)	<0.001	1.11
Symmetry/Ordering	7.18 (6.17)	3.51 (4.03)	<0.001	0.85
Contamination/Cleaning	5.03 (6.24)	2.45 (4.04)	<0.001	0.59
Body dysmorphic	9.47 (7.01)	6.27 (5.62)	<0.001	0.55
Hoarding	4.31 (4.89)	2.34 (3.32)	<0.001	0.56
Hair-pulling	3.72 (6.16)	1.14 (3.44)	<0.001	0.67
Skin-picking	4.61 (5.68)	2.46 (3.83)	<0.001	0.53
	**HPD/SPD,** ***M* (*SD*)**	**No HPD/SPD,** ***M* (*SD*)**	***p* for difference**	**Cohen’s *d***
Harm/Checking	5.31 (5.26)	3.04 (3.94)	<0.001	0.55
Taboo obsessions	3.24 (4.88)	1.60 (3.37)	<0.001	0.46
Symmetry/Ordering	5.93 (5.82)	3.72 (4.26)	<0.001	0.50
Contamination/Cleaning	2.81 (4.45)	2.73 (4.42)	0.87	0.02
Body dysmorphic	10.10 (6.79)	6.28 (5.67)	<0.001	0.66
Hoarding	3.31 (4.57)	2.48 (3.46)	0.14	0.23
Hair-pulling	8.03 (7.50)	0.76 (2.52)	<0.001	2.20
Skin-picking	6.23 (6.26)	2.33 (3.66)	<0.001	0.98

Group differences were examined using Mann–Whitney *U* tests. HPD, hair-pulling disorder; OCD, obsessive-compulsive disorder; OCRD-D-E, obsessive-compulsive and related disorders-dimensional scales-expanded version; SPD, skin-picking disorder.

### Factor structure of OCD dimensions and related disorders

The correlations (standardized covariance coefficients) in the CFA between the eight OCRD-D-E factors ranged from 0.11 (contamination/cleaning and HPD) to 0.54 (disturbing thoughts/checking and symmetry/ordering *plus* disturbing thoughts/checking and taboo obsessions) indicating that overarching factor structures may be present. We split the sample into random halves and conducted EFA with the first half of the sample. First, we estimated a correlation matrix for the eight OCRD-D-E domains using the non-parametric latent Gaussian copula model. The overall KMO value for the eight variables was 0.83 and no single KMO value was under 0.78, indicating that the data were suitable for EFA. The Bartlett’s test of sphericity was significant (*p* < 0.001), further indicating that the data were well-suited for EFA. Parallel analysis suggested 4 factors, which were extracted using principal axis factoring and promax rotation. The four factors explained 43.7% of the covariance among the eight domains. The proposed factor structure indicated that harm/checking and taboo obsessions loaded onto a common factor. SPD and HPD did also load onto a common factor as well as symmetry/ordering and contamination/cleaning, although the latter factor was somewhat problematic since the loading of symmetry/ordering was 1.04 while the loading of contamination/cleaning was only 0.39, which may indicate that the two variables are best considered separate factors. This possibility was tested in the CFA phase.

**FIGURE 1:**
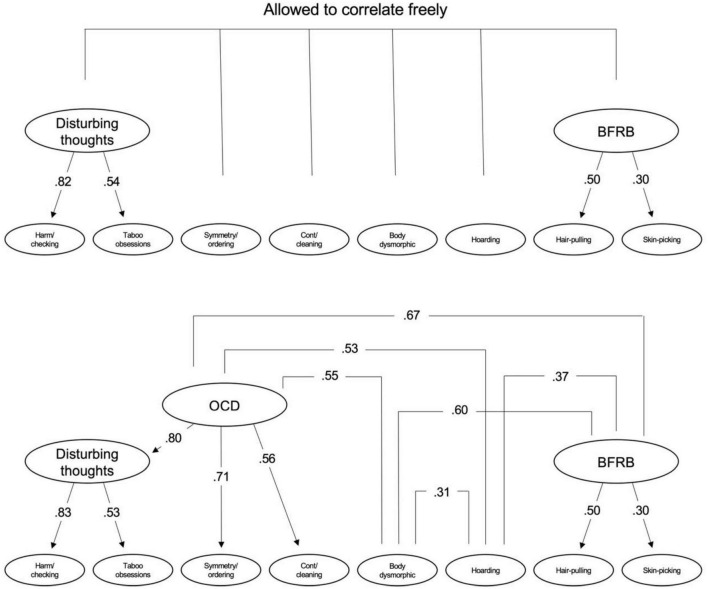
At the **top** is a model where the empirically derived factors were allowed to correlate freely and at the **bottom** is a model where an overarching OCD factor is included which then is allowed to correlate freely with the rest of the factors. Correlations among the factors in the model at the top is presented in Table 4 and correlations among the factors in the model at the bottom are presented in this figure. BFRB, body-focused repetitive behaviors; OCD, obsessive-compulsive disorder.

We fitted two models in the second half of the sample. In both models, harm/checking and taboo thoughts and hair-pulling and skin-picking were included under common factors, while BDD and hoarding were included as separate factors. In the first model, we included symmetry/ordering and contamination/cleaning under a common factor, while in the second model these two domains were included as separate factors. The second model showed better model/data fit than the first model: first model, CFI = 0.98, TLI = 0.95, RMSEA = 0.05, SRMR = 0.03; second model, CFI = 0.99, TLI = 0.97, RMSEA = 0.04, SRMR = 0.02. Because of its theoretical merit, and to examine relations among the new factors, we fitted a model that grouped the three OCD factors under a second-order factor. Compared to the model where all factors were allowed to correlate freely, the model/data fit for the model with a second-order OCD factor was somewhat poorer but still adequate (CFI = 0.98, TLI = 0.95, RMSEA = 0.05, SRMR = 0.03). The two models are presented graphically in Figure 1. Correlations (standardized covariance coefficients) among the factors for the model with the second-order OCD factor are in Figure 1; correlations among the factors for the model where all factors were allowed to correlate freely are in Table 4.

**TABLE 4:** CFA-based standardized covariance coefficients among the OCRD-D-E factors.

	1	2	3	4	5	6
1. Disturbing thoughts	–					
2. Symmetry/Ordering	0.55	–				
3. Contamination/Cleaning	0.45	0.43	–			
4. Body-dysmorphic symptoms	0.45	0.40	0.29	–		
5. Hoarding	0.45	0.36	0.30	0.31	–	
6. Body-focused repetitive behaviors	0.63	0.55	0.19	0.61	0.38	–

All covariance coefficients are significant at the *p* < 0.001 level. CFA, confirmatory factor analysis; OCRD-D-E, obsessive-compulsive and related disorders-dimensional scales-expanded version.

## Discussion

We expanded a DSM-5-based dimensional self-report measure of HD, BDD, HPD, and SPD to include the major symptom dimensions of OCD. This updated and comprehensive measure showed excellent psychometric properties, adequate test-retest reliability, known groups validity, and expected associations with well-being, depression/anxiety, and satisfaction with life. All domains of the measure correlated negatively with satisfaction with life and well-being and positively with anxiety/depression, with the BDD and harm/checking dimensions showing the strongest associations with external validators. These findings are in line with results of a recent meta-analysis that found higher rates of suicidality in BDD compared to the other related disorders (49), supporting the presence of poorer mental health in people who present with BDD symptoms. In contrast, the dimension with the lowest associations with satisfaction with life, depression/anxiety, and well-being was the contamination/cleaning dimension. This is also in line with previous study showing that harm/checking symptoms in OCD are more clearly related to anxiety and depression than the contamination/cleaning and symmetry/ordering factors (14).

Using exploratory and confirmatory factor analysis, we found that harm/checking and taboo obsessions formed a common factor. This is consistent with a recent study where the OCD dimensions of harm/checking and taboo obsessions formed a higher-order factor which was termed *disturbing thoughts* to indicate the clear cognitive involvement (often in the form of intrusive thoughts) in symptoms (14). We used the same term for this factor in the present study.

Regarding relations among all OCRD domains, the present study presents the most comprehensive examination of such relations since it includes the heterogeneity of OCD and measures all OCRD dimensions/disorders using the same severity items. Results are only partially in line with prior research [e.g., (5, 50)], which has indicated that these disorders can be organized under two broad factors: an “obsessions-compulsions” factor composed of the “cognitive” (i.e., an emphasis on intrusive thoughts) symptom dimensions (OCD, BDD, and HD) and a “body-focused repetitive behaviors” (BFRB) factor (which is proposed to lack a prominent cognitive component) that includes SPD and HPD (5). Prior research has shown that the “cognitive” factor is genetically correlated with anxiety disorder symptoms (51) and it has been proposed that all of these symptom dimensions are different expressions of internalizing psychopathology (50). In the present study, both the disturbing thoughts and symmetry/ordering OCD factors were more strongly associated with BFRB than with BDD and hoarding. Similarly, the overarching OCD factor that included all OCD factors was also more strongly associated with BFRB than with BDD and hoarding. These results do not support an overarching cognitive factor. We hope that the availability of a new measure that makes it possible to simultaneously assess the full spectrum of OCD and related disorders can stimulate research on how these disorders relate to each other and to other psychiatric disorders, for example, anxiety and psychosis spectrum disorders (52).

Regarding HPD and SPD, our results indicate that they load onto a shared BFRB factor, which is in line with findings showing that all the genetic variance of a second factor is shared between HPD and SPD and that such symptoms may be phenotypic expressions of the same underlying condition (5). As mentioned in the introduction, the diagnostic placement of HPD and SPD in the OCD chapter has been debated (11). Both disorders show many similarities in a variety of clinical features, including symptom expression (53). However, as highlighted by Snorrason et al. (50), the disorders differ in relevant ways when compared with the other disorders in the OCRD chapter since they seem to lack a clear obsessive (intrusive thoughts) component. Snorrason et al. (54, 55) found that there are two characteristic processes in HPD and SPD: (1) appetitive urges to engage in repetitive and/or compulsive behaviors such as hair pulling in HPD or skin scratching in SPD and (2) pleasure or gratification while performing the behaviors. In contrast, in OCD and BDD, repetitive/compulsive behaviors are often associated with unpleasant antecedent internal events (worry, fear, disgust, etc.) (33). In HD, there may be a component of pleasure or satisfaction in symptoms (56), but this is not as immediate as in HPD and SPD. In addition, some authors [e.g., (57, 58)] propose alternative ways of classifying impulsivity and compulsivity, where BFRB are argued to belong under an impulsivity dimension in contrast to other dimensions/disorders that are assigned to the compulsivity dimension. Additionally, previous research has found relations between BFRBs and addictive disorders, from similarity in their characteristics, pharmacological treatment (59–61) and family studies (62). The present study can provide no final conclusions about the above controversies, but supports that HPD and SPD are more closely related to each other than to OCD, BDD, and HD. Our results also support that all dimensions/disorders are positively associated, which may imply common underlying vulnerabilities or similar causing and/or maintaining mechanisms. To some degree the present results indicate that the major symptom dimensions of OCD can be divided into a more cognitive factor revolving around intrusive and anxiety-provoking thoughts (harm/checking and taboo obsessions) and a more compulsive factor (symmetry/ordering and contamination/cleaning), although the two latter dimensions were best considered separate. A division of OCD symptoms into overarching cognitive/anxious and compulsive factors is in line with the notion that compulsivity and anxiety are the two major underlying processes in OCD [e.g., (63)].

The OCRD-D-E appears to adequately assess the major symptom dimensions of OCD and related disorders using a single self-report scale based on the suggested dimensionality of the DSM-5. Moreover, the scale is time-efficient and freely available. Thus, it can have both clinical and research utility, but more work is needed on construct validity, sensitivity to change, clinimetric properties, and incremental validity. For construct validity, studies should examine to which degree the scales correlate with other OCRD measures; further, classification performance of the OCRD-D-E in relation to interview-based diagnoses should be examined. Sensitivity to change is important to examine if the scale is intended to be used in clinical practice and such research may be best carried out within the context of clinical trials or other studies where structured treatment and treatment outcome assessments are implemented (a 7 days’ time frame is probably best if used as an outcome measure). Clinimetric properties refer to the ability of a measure to capture information that is important for patient care and for patients themselves (e.g., real-life change, impairment, etc.), and future studies should secure that broad aspects of relevance for individuals with OCD and related disorders are captured during assessment (64). Given that many measures are available for OCRD disorders, it is important to conduct research on the incremental validity of the OCRD-D-E, that is, if it adds unique information (e.g., related to prognosis, diagnostic sensitivity/specificity) relative to other available measures.

This study has some limitations. First, we relied solely on self-report and future studies should use clinical interviews which also would facilitate research on sensitivity/specificity in relation to diagnostic status. Second, the study was conducted using an online survey and thus it is unclear to which population it is valid to generalize the findings. Future research should include well-defined clinical participants. Third, the measure is dependent upon the broad vignettes of each dimension, which were based on previous scales, but the validity of these vignettes needs to be examined. Fourth, this study did not examine factorial invariance across gender or age groups; although it was not an objective of the study itself, it could have affected the results. Fifth, to provide more definitive results about the validity of the new OCRD chapter, the domains/disorders analyzed in the present study need to be assessed alongside valid information on other relevant psychiatric symptom dimensions such as anxiety disorders, major depression, eating disorders, and psychosis spectrum disorders.

Despite these limitations, this work represents a first approach to a unified way to assess OCD and related disorders using a single and dimensional self-report scale. The updated scale has strong psychometric properties which supports its use in clinical practice and research. The scale provides clinicians and researchers with the ability to comprehensively assess OCD and related disorders, including the different OCD symptom dimensions, making it possible to examine comorbidity profiles and relations between these disorders, as well as to inform genetic and family history studies. The results did not support the presence of two overarching factors that include OCD/BDD/HD (“obsessions-compulsions” factor) and HPD/SPD (BFRB factor). While HPD and SPD appear to relate more strongly to each other than to the other domains/disorders, no clear overarching “obsessions-compulsions” factor emerged. However, all factors were positively correlated indicating that mutual vulnerability/underlying mechanisms may be in play. Future research is needed to further explore the psychometric properties of the OCRD-D-E and whether it can be used to improve our understanding of OCD and related disorders across the lifespan. Despite some initial results regarding overarching factor structures, as for now, we recommend using all eight modules and interpreting them independently.

## Data availability statement

The raw data supporting the conclusions of this article will be made available by the authors, without undue reservation.

## Ethics statement

The studies involving human participants were reviewed and approved by Project Evaluation Committee from the Miguel Hernández University (DPS.JPR.03.20). Written informed consent to participate in this study was provided by the participants’ legal guardian/next of kin.

## Author contributions

BM-A participated in the conceptualization of the study, in the selection and adaptation of the instruments, and design of the assessment protocol, coordinated and supervised the data collection, managed the database and literature review tasks, drafted the initial manuscript, and reviewed the contents of the manuscript and adapted its presentation format to the formatting requirements. JP was principal investigator of the funded project, participated in the design of the psychological assessment protocol, collaborated in the conceptualization of this study by providing theoretical knowledge and professional and research background, and carried out a review of the content. TR-J and AM-G participated in the conceptualization of the study, drafted the initial introduction of the manuscript, assisted in literature review tasks, and reviewed the contents and adapted its presentation format to the formatting requirements. MC participated in the conceptualization of the study by providing theoretical knowledge and professional and research background, provided an analytical-methodological perspective, undertook the analysis of the data that make up the results of the study, and carried out a review of the content. All authors approved the final manuscript as submitted and agreed to be accountable for all aspects of the work.
