# Supplementary material for: Measuring symptoms of obsessive-compulsive and related disorders using a single dimensional self-report scale
Source: Front Psychiatry. 2023 Feb 14;14:958015. doi: 10.3389/fpsyt.2023.958015 (PMC9971505; doi:10.3389/fpsyt.2023.958015)
Supplement: Supplementary file 1 [file Table_1.DOCX]

Supplemental information

**OCRD-D-E: SPANISH VERSION**

**A. Pensamientos/comportamientos relacionados con hacer daño o ser responsable de algo malo y conductas de comprobación**

¿Alguna vez has tenido pensamientos, ideas o imágenes molestas sobre hacer daño a otras personas, posibles eventos catastróficos o tener la responsabilidad de que algo de esto suceda? Por ejemplo, pensamientos sobre algo malo que te suceda a ti mismo o a otra persona, o sobre dañar a otras personas o ser responsable del daño que sufran otras personas. A menudo, estos pensamientos vienen en forma de "¿y si ...?" y están acompañados de conductas de comprobación u otro tipo de conductas o actos mentales realizados para prevenir o controlar posibles daños. Estas conductas pueden ser: *verificar que la puerta o las ventanas estén cerradas, o los enchufes protegidos*. A veces tienes que realizar estas conductas una y otra vez. También es común buscar la reaseguración a partir de otras personas (p.e. padres, amigos, pareja), por ejemplo, preguntándoles para asegurarte que realmente cerraste la puerta, la ventana, etc. Además, es frecuente evitar situaciones, objetos o personas que provoquen estos pensamientos.

A1. ¿Alguna vez has tenido este tipo de pensamientos/comportamientos? (Marca la respuesta)

**Sí No**

A2. ¿Has tenido este tipo de pensamientos/comportamientos durante **EL ÚLTIMO MES**?

(Si no has tenido dificultades, Marca 0 como opción de respuesta)

| En término medio, ¿con qué *frecuencia* te han ocurrido estos pensamientos/  comportamientos cada día? | **0**  Nada | **1**  Un poco (ocasionalmente) | **2**  Bastante  (a menudo) | **3**  Mucho (muy a menudo) | **4**  Muchísimo (casi siempre) |
| --- | --- | --- | --- | --- | --- |
| ¿Cuánto *malestar* te han causado estos pensamientos/  comportamientos? | **0**  Ninguno | **1**  Un poco | **2**  Bastante | **3**  Mucho | **4**  Muchísimo (me incapacita) |
| ¿Cuánto te ha costado *controlar estos* pensamientos/  comportamientos? | **0**  Nada (tengo un control completo) | **1**  Un poco (tengo mucho control) | **2**  Bastante (tengo control moderado) | **3**  Mucho (tengo poco control) | **4**  Muchísimo (no tengo ningún control) |
| ¿Estos pensamientos/  comportamientos han hecho que *evites o dejes de* hacer algo, de ir a algún lugar o de estar con alguien? | **0**  Nunca (ninguna evitación) | **1**  Pocas veces (evitación ocasional) | **2**  Algunas veces (evitación moderada) | **3**  Muchas veces (evitación frecuente y amplia) | **4**  Siempre (evitación extrema: soy incapaz de salir de casa) |
| ¿Cuánto han interferido *(te limitan)* estos pensamientos/  comportamientos en tu vida diaria (en tu vida escolar/académica, laboral, social o familiar)? | **0**  Nada (ninguna interferencia) | **1**  Un poco (ligera interferencia) | **2**  Bastante (interferencia moderada; definitivamente interfiere en mi vida) | **3**  Mucha interferencia | **4**  Muchísimo (interferencia extrema; me limita e incapacita) |

**B. Pensamientos prohibidos sobre la sexualidad, la religión y la moralidad**

¿Alguna vez ha tenido pensamientos molestos que giran en torno a temas sexuales, religiosos o morales? Los pensamientos religiosos pueden ser sobre blasfemia, violación de reglas o costumbres religiosas y pueden causar la necesidad de confesar o rezar. Los pensamientos sexuales pueden ser sobre orientación sexual, prácticas sexuales (por ejemplo, sexo con niños o animales) o imágenes mentales de escenas o actos sexuales angustiantes. Los pensamientos morales pueden ser acerca de hacer algo que es moralmente incorrecto o moralmente reprensible/malo. Este tipo de pensamientos a menudo se consideran tabú o pensamientos prohibidos y es común evitar situaciones, objetos o personas que los desencadenan.

B1. ¿Alguna vez has tenido este tipo de pensamientos? (Marca la respuesta)

**Sí No**

B2. ¿Has tenido este tipo de pensamientos durante **EL ÚLTIMO MES**?

(Si no has tenido dificultades, Marca 0 como opción de respuesta)

| En término medio, ¿con qué *frecuencia* te han ocurrido estos pensamientos cada día? | **0**  Nada | **1**  Un poco (ocasionalmente) | **2**  Bastante  (a menudo) | **3**  Mucho (muy a menudo) | **4**  Muchísimo (casi siempre) |
| --- | --- | --- | --- | --- | --- |
| ¿Cuánto *malestar* te han causado estos pensamientos? | **0**  Ninguno | **1**  Un poco | **2**  Bastante | **3**  Mucho | **4**  Muchísimo (me incapacita) |
| ¿Cuánto te ha costado *controlar estos* pensamientos? | **0**  Nada (tengo un control completo) | **1**  Un poco (tengo mucho control) | **2**  Bastante (tengo control moderado) | **3**  Mucho (tengo poco control) | **4**  Muchísimo (no tengo ningún control) |
| ¿Estos pensamientos han hecho que *evites o dejes de* hacer algo, de ir a algún lugar o de estar con alguien? | **0**  Nunca (ninguna evitación) | **1**  Pocas veces (evitación ocasional) | **2**  Algunas veces (evitación moderada) | **3**  Muchas veces (evitación frecuente y amplia) | **4**  Siempre (evitación extrema: soy incapaz de salir de casa) |
| ¿Cuánto han interferido *(te limitan)* estos pensamientos en tu vida diaria (en tu vida escolar/académica, laboral, social o familiar)? | **0**  Nada (ninguna interferencia) | **1**  Un poco (ligera interferencia) | **2**  Bastante (interferencia moderada; definitivamente interfiere en mi vida) | **3**  Mucha interferencia | **4**  Muchísimo (interferencia extrema; me limita e incapacita) |

**C. Pensamientos/comportamientos sobre simetría, sensaciones del tipo “algo no está bien del todo, o como se supone que debería estar para decir que «ya está correcto» o «finalizado»”, y conductas repetitivas y de orden**

¿Alguna vez has tenido pensamientos o sensaciones molestas sobre la necesidad de tener las cosas niveladas, equilibradas, simétricas o uniformes? Estos pensamientos a menudo están relacionados con que los objetos, el propio cuerpo o ciertas acciones se experimentan como "no están como tienen que estar" o "están/son incompletas". Los pensamientos y sensaciones pueden llevarte a repetir acciones, ordenar cosas, tocar cosas de cierta manera, o arreglar las cosas. También es común contar o realizar conductas para conseguir la sensación de que las cosas, o el propio cuerpo, estén equilibradas o tengan una determinada textura y suavidad. Es frecuente evitar situaciones y objetos que provocan esos pensamientos/comportamientos/sensaciones molestas.

C1. ¿Alguna vez has tenido este tipo de pensamientos/comportamientos/sensaciones? (Marca la respuesta)

**Sí No**

C2. ¿Has tenido este tipo de pensamientos/comportamientos/sensaciones durante **EL ÚLTIMO MES**?

(Si no has tenido dificultades, Marca 0 como opción de respuesta)

| En término medio, ¿con qué *frecuencia* te han ocurrido estos pensamientos/  comportamientos/  sensaciones cada día? | **0**  Nada | **1**  Un poco (ocasionalmente) | **2**  Bastante  (a menudo) | **3**  Mucho (muy a menudo) | **4**  Muchísimo (casi siempre) |
| --- | --- | --- | --- | --- | --- |
| ¿Cuánto *malestar* te han causado estos pensamientos/  comportamientos/  sensaciones? | **0**  Ninguno | **1**  Un poco | **2**  Bastante | **3**  Mucho | **4**  Muchísimo (me incapacita) |
| ¿Cuánto te ha costado *controlar estos* pensamientos/  Comportamientos/  sensaciones? | **0**  Nada (tengo un control completo) | **1**  Un poco (tengo mucho control) | **2**  Bastante (tengo control moderado) | **3**  Mucho (tengo poco control) | **4**  Muchísimo (no tengo ningún control) |
| ¿Estos pensamientos/  Comportamientos/sensaciones han hecho que *evites o dejes de* hacer algo, de ir a algún lugar o de estar con alguien? | **0**  Nunca (ninguna evitación) | **1**  Pocas veces (evitación ocasional) | **2**  Algunas veces (evitación moderada) | **3**  Muchas veces (evitación frecuente y amplia) | **4**  Siempre (evitación extrema: soy incapaz de salir de casa) |
| ¿Cuánto han interferido *(te limitan)* estos pensamientos/  Comportamientos/sensaciones en tu vida diaria (en tu vida escolar/académica, laboral, social o familiar)? | **0**  Nada (ninguna interferencia) | **1**  Un poco (ligera interferencia) | **2**  Bastante (interferencia moderada; definitivamente interfiere en mi vida) | **3**  Mucha interferencia | **4**  Muchísimo (interferencia extrema; me limita e incapacita) |

**D. Pensamientos/comportamientos relacionados con contaminación, suciedad, asco y comportamientos de limpieza.**

¿Alguna vez ha tenido pensamientos, ideas o imágenes sobre contaminación, gérmenes y limpieza?

*Para responder a esta pregunta, piensa sobre tus pensamientos en general, y no únicamente en aquellos debido a la situación excepcional actual sobre la pandemia de la Covid-19 donde estos pensamientos y comportamientos de higiene y distancia física/social son medidas sanitarias asumidas por toda la sociedad.*

Tales pensamientos pueden llevar a la sensación de que necesitas lavarte las manos u otras partes del cuerpo, limpiar tus cosas o separar las cosas "sucias" de las cosas "limpias". Los pensamientos pueden hacer que te cueste tocar cosas que otras personas han tocado. Además, esto puede hacer que gastes mucho tiempo y energía en limpiarte a ti mismo o a tus cosas. Ir al baño puede llevarte mucho tiempo y puede implicar ciertos comportamientos compulsivos y evitar ciertos objetos. Los pensamientos pueden implicar miedo de ser infectado, a ser envenenado, a infectarte por gérmenes o a contagiar o propagar una enfermedad. Algunas personas sólo sienten asco. Todo esto puede hacer que te cueste manipular o estar cerca de productos químicos o entrar en contacto con sustancias pegajosas. Es común evitar situaciones, objetos o personas que provoquen estos pensamientos/comportamientos.

D1. ¿Alguna vez has tenido este tipo de pensamientos/comportamientos? (Marca la respuesta)

**Sí No**

D2. ¿Has tenido este tipo de pensamientos/comportamientos durante **EL ÚLTIMO MES**?

(Si no has tenido dificultades, Marca 0 como opción de respuesta)

| En término medio, ¿con qué *frecuencia* te han ocurrido estos pensamientos/  comportamientos cada día? | **0**  Nada | **1**  Un poco (ocasionalmente) | **2**  Bastante  (a menudo) | **3**  Mucho (muy a menudo) | **4**  Muchísimo (casi siempre) |
| --- | --- | --- | --- | --- | --- |
| ¿Cuánto *malestar* te han causado estos pensamientos/  comportamientos? | **0**  Ninguno | **1**  Un poco | **2**  Bastante | **3**  Mucho | **4**  Muchísimo (me incapacita) |
| ¿Cuánto te ha costado *controlar estos* pensamientos/  comportamientos? | **0**  Nada (tengo un control completo) | **1**  Un poco (tengo mucho control) | **2**  Bastante (tengo control moderado) | **3**  Mucho (tengo poco control) | **4**  Muchísimo (no tengo ningún control) |
| ¿Estos pensamientos/  comportamientos han hecho que *evites o dejes de* hacer algo, de ir a algún lugar o de estar con alguien? | **0**  Nunca (ninguna evitación) | **1**  Pocas veces (evitación ocasional) | **2**  Algunas veces (evitación moderada) | **3**  Muchas veces (evitación frecuente y amplia) | **4**  Siempre (evitación extrema: soy incapaz de salir de casa) |
| ¿Cuánto han interferido *(te limitan)* estos pensamientos/  comportamientos en tu vida diaria (en tu vida escolar/académica, laboral, social o familiar)? | **0**  Nada (ninguna interferencia) | **1**  Un poco (ligera interferencia) | **2**  Bastante (interferencia moderada; definitivamente interfiere en mi vida) | **3**  Mucha interferencia | **4**  Muchísimo (interferencia extrema; me limita e incapacita) |

**E. Preocupaciones/comportamientos relacionados con defectos en la apariencia física**

¿Alguna vez has tenido inquietudes o preocupaciones sobre tu apariencia física (defectos que percibes sobre tu apariencia) o comportamientos repetitivos que realizas en respuesta a estas inquietudes/preocupaciones?

Estas inquietudes/preocupaciones pueden estar relacionadas con uno o más defectos que tú percibes en tu apariencia física y que experimentas como muy perturbadores/molestos, pero que no son en absoluto observables para las demás personas o solo lo son ligeramente. Es común realizar comportamientos repetitivos (por ejemplo, mirarse en el espejo, arreglarse demasiado, pellizcarse la piel o preguntar a otras personas sobre esos defectos) o actos mentales (por ejemplo, comparar tu apariencia con la de otras personas) en respuesta a estas preocupaciones sobre la apariencia. También es común evitar situaciones, objetos o personas debido a estas preocupaciones/comportamientos y tratar de ocultar los defectos percibidos cuando hay otras personas presentes.

Las inquietudes/preocupaciones no deben estar relacionadas únicamente con el peso o la grasa corporal.

E1. ¿Alguna vez has tenido este tipo de preocupaciones/comportamientos? (Marca la respuesta)

**Sí No**

E2. ¿Has tenido este tipo de preocupaciones/comportamientos durante **EL ÚLTIMO MES**? (Si no has tenido dificultades, Marca 0 como opción de respuesta)

| En término medio, ¿con qué *frecuencia* te han ocurrido estas preocupaciones/  comportamientos cada día? | **0**  Nada | **1**  Un poco (ocasionalmente) | **2**  Bastante  (a menudo) | **3**  Mucho (muy a menudo) | **4**  Muchísimo (casi siempre) |
| --- | --- | --- | --- | --- | --- |
| ¿Cuánto *malestar* te han causado estas preocupaciones/  comportamientos? | **0**  Ninguno | **1**  Un poco | **2**  Bastante | **3**  Mucho | **4**  Muchísimo (me incapacita) |
| ¿Cuánto te ha costado *controlar* estas preocupaciones/  comportamientos? | **0**  Nada (tengo un control completo) | **1**  Un poco (tengo mucho control) | **2**  Bastante (tengo control moderado) | **3**  Mucho (tengo poco control) | **4**  Muchísimo (no tengo ningún control) |
| ¿Estas preocupaciones/  comportamientos han hecho que *evites o dejes de* hacer algo, de ir a algún lugar o de estar con alguien? | **0**  Nunca (ninguna evitación) | **1**  Pocas veces (evitación ocasional) | **2**  Algunas veces (evitación moderada) | **3**  Muchas veces (evitación frecuente y amplia) | **4**  Siempre (evitación extrema: soy incapaz de salir de casa) |
| ¿Cuánto han interferido *(te limitan)* estas preocupaciones/  comportamientos en tu vida diaria (en tu vida escolar/académica, laboral, social o familiar)? | **0**  Nada (ninguna interferencia) | **1**  Un poco (ligera interferencia) | **2**  Bastante (interferencia moderada; definitivamente interfiere en mi vida) | **3**  Mucha interferencia | **4**  Muchísimo (interferencia extrema; me limita e incapacita) |

**F. Dificultades/comportamientos relacionados con la acumulación de cosas**

¿Alguna vez has tenido una dificultad persistente para deshacerte o desprenderte de cosas que tienes, independientemente de su valor real, porque sientes una gran necesidad de guardar estas cosas y/o porque sentirías angustia si tratas de deshacerte de ellas?

Esta dificultad para deshacerse de cosas a menudo provoca la acumulación de un gran número de objetos que congestionan y abarrotan ciertos espacios del lugar donde se vive hasta el punto de que dificulta vivir en ese lugar. Además, es común recoger, comprar o robar cosas que no son necesarias o para las que no hay espacio disponible para guardarlas.

F1. ¿Alguna vez has acumulado cosas como se describe anteriormente? (Marca la respuesta)

**Sí No**

F2. ¿Has acumulado cosas como se describe anteriormente durante **EL ÚLTIMO MES**?

(Si no has tenido dificultades, Marca 0 como opción de respuesta)

| ¿Hasta qué punto tienes *dificultad para deshacerte* (tirar, reciclar, vender, regalar) cosas ordinarias de las que otras personas se desharían? | **0**  Nada (ninguna dificultad) | **1**  Un poco | **2**  Bastante | **3**  Mucho | **4**  Muchísimo |
| --- | --- | --- | --- | --- | --- |
| ¿Cuánto *malestar* te han causado estas preocupaciones/  comportamientos? | **0**  Ninguno | **1**  Un poco | **2**  Bastante | **3**  Mucho | **4**  Muchísimo (me incapacita) |
| Debido al desorden o la cantidad de cosas, ¿hasta qué punto te resulta *difícil usar* los espacios y habitaciones de tu casa? | **0**  Nada (ninguna dificultad) | **1**  Un poco | **2**  Bastante | **3**  Mucho | **4**  Muchísimo |
| ¿Estas preocupaciones/  comportamientos han hecho que *evites o dejes de* hacer algo, de ir a algún lugar o de estar con alguien? | **0**  Nunca (ninguna evitación) | **1**  Pocas veces (evitación ocasional) | **2**  Algunas veces (evitación moderada) | **3**  Muchas veces (evitación frecuente y amplia) | **4**  Siempre (evitación extrema: soy incapaz de salir de casa) |
| ¿Cuánto han interferido *(te limitan)* estas preocupaciones/  comportamientos en tu vida diaria (en tu vida escolar/académica, laboral, social o familiar)? | **0**  Nada (ninguna interferencia) | **1**  Un poco (ligera interferencia) | **2**  Bastante (interferencia moderada; definitivamente interfiere en mi vida) | **3**  Mucha interferencia | **4**  Muchísimo (interferencia extrema; me limita e incapacita) |

**G. Ganas/conductas relacionadas con arrancarse pelo**

¿Alguna vez has tenido **ganas, el impulso o la necesidad**de arrancarte el pelo de la cabeza o de cualquier otra parte del cuerpo y/o alguna vez te has arrancado el pelo de la cabeza / otra parte del cuerpo (sin el propósito de hacerte daño ni por motivos estéticos)?

Arrancarse el pelo se caracteriza por arrancarse el pelo de forma recurrente dando como resultado la pérdida de pelo, e intentar repetidamente disminuir o dejar de arrancarse el pelo. Esta conducta no está causada ​​por pensamientos angustiantes específicos o preocupaciones sobre la apariencia; sin embargo, puede estar precedida o acompañada de diversos estados emocionales, como sentimientos de ansiedad, estrés o aburrimiento. Esta conducta también puede estar precedida por una creciente sensación de tensión y tirar del pelo puede conducir a gratificación, placer o una sensación de alivio. Es común tener diversos grados de conciencia de la conducta mientras se realiza. A veces se puede ser muy consciente de la conducta (con tensión precedente y alivio posterior), otras veces la conducta puede realizarse sin conciencia.

Es común evitar situaciones, objetos o personas debido a estas ganas, debido a la conducta de arrancar pelo y/o por la pérdida resultante de pelo.

G1. ¿Alguna vez has tenido este tipo de ganas, impulso o necesidad/conductas? (Marca la respuesta)

**Sí No**

G2. ¿Has tenido este tipo de ganas, impulso o necesidad/conductas durante **EL ÚLTIMO MES**?

(Si no has tenido dificultades, Marca 0 como opción de respuesta)

| En término medio, ¿con qué *frecuencia* has tenido estas ganas, impulso o necesidad/  conductas cada día? | **0**  Nada | **1**  Un poco (ocasionalmente) | **2**  Bastante (a menudo) | **3**  Mucho (muy a menudo) | **4**  Muchísimo (casi siempre) |
| --- | --- | --- | --- | --- | --- |
| ¿Cuánto *malestar* te han causado estas ganas, impulso o necesidad/conductas? | **0**  Ninguno | **1**  Un poco | **2**  Bastante | **3**  Mucho | **4**  Muchísimo (me incapacita) |
| ¿Cuánto te ha costado *controlar* estas ganas, impulso o necesidad/conductas? | **0**  Nada (tengo un control completo) | **1**  Un poco (tengo mucho control) | **2**  Bastante (tengo control moderado) | **3**  Mucho (tengo poco control) | **4**  Muchísimo (no tengo ningún control) |
| ¿Estas ganas, impulso o necesidad/conductas han hecho que *evites o dejes de* hacer algo, de ir a algún lugar o de estar con alguien? | **0**  Nunca (ninguna evitación) | **1**  Pocas veces (evitación ocasional) | **2**  Algunas veces (evitación moderada) | **3**  Muchas veces (evitación frecuente y amplia) | **4**  Siempre (evitación extrema: soy incapaz de salir de casa) |
| ¿Cuánto han interferido *(te limitan)* estas ganas, impulso o necesidad/  conductas en tu vida diaria (en tu vida escolar/ académica, laboral, social o familiar)? | **0**  Nada (ninguna interferencia) | **1**  Un poco (ligera interferencia) | **2**  Bastante (interferencia moderada; definitivamente interfiere en mi vida) | **3**  Mucha interferencia | **4**  Muchísimo (interferencia extrema; me limita e incapacita) |

**H. Ganas/conductas relacionadas con pellizcarse/rascarse la piel**

¿Alguna vez has tenido ganas, impulso o necesidad de pellizcarte/rascarte la piel o te has pellizcado/rascarse la piel (sin el propósito de hacerte daño, ni por motivos estéticos)? El pellizcarse la piel se caracteriza por pellizcar, rascar, arrancar y/o hurgar imperfecciones de tu piel (como, por ejemplo: heridas, granos, espinillas, poros, etc.) de forma recurrente, lo que da como resultado lesiones cutáneas, e intentar repetidamente disminuir o dejar de pellizcarse. Esta conducta no está causada ​​por pensamientos angustiantes específicos o preocupaciones sobre la apariencia; sin embargo, puede estar precedida o acompañada de diversos estados emocionales, como sentimientos de ansiedad, estrés o aburrimiento. Esta conducta también puede estar precedida por una creciente sensación de tensión y pellizcar la piel puede conducir a gratificación, placer o una sensación de alivio. Es común tener diversos grados de conciencia de la conducta mientras se realiza. A veces se puede ser muy consciente de la conducta (con tensión precedente y alivio posterior), otras veces la conducta puede realizarse sin conciencia.

Es común evitar situaciones, objetos o personas debido a estas ganas, debido a la conducta de pellizcar la piel y/o por las heridas cutáneas resultantes.

H1. ¿Alguna vez has tenido este tipo de ganas, impulso o necesidad/conductas? (Marca la respuesta)

**Sí No**

H2. ¿Has tenido este tipo de ganas, impulso o necesidad/conductas durante **EL ÚLTIMO MES**?

(Si no has tenido dificultades, Marca 0 como opción de respuesta)

| En término medio, ¿con qué *frecuencia* has tenido estas ganas, impulso o necesidad/  conductas cada día? | **0**  Nada | **1**  Un poco (ocasionalmente) | **2**  Bastante (a menudo) | **3**  Mucho (muy a menudo) | **4**  Muchísimo (casi siempre) |
| --- | --- | --- | --- | --- | --- |
| ¿Cuánto *malestar* te han causado estas ganas, impulso o necesidad/conductas? | **0**  Ninguno | **1**  Un poco | **2**  Bastante | **3**  Mucho | **4**  Muchísimo (me incapacita) |
| ¿Cuánto te ha costado *controlar* estas ganas, impulso o necesidad/conductas? | **0**  Nada (tengo un control completo) | **1**  Un poco (tengo mucho control) | **2**  Bastante (tengo control moderado) | **3**  Mucho (tengo poco control) | **4**  Muchísimo (no tengo ningún control) |
| ¿Estas ganas, impulso o necesidad/conductas han hecho que *evites o dejes de* hacer algo, de ir a algún lugar o de estar con alguien? | **0**  Nunca (ninguna evitación) | **1**  Pocas veces (evitación ocasional) | **2**  Algunas veces (evitación moderada) | **3**  Muchas veces (evitación frecuente y amplia) | **4**  Siempre (evitación extrema: soy incapaz de salir de casa) |
| ¿Cuánto han interferido *(te limitan)* estas ganas, impulso o necesidad/  conductas en tu vida diaria (en tu vida escolar/ académica, laboral, social o familiar)? | **0**  Nada (ninguna interferencia) | **1**  Un poco (ligera interferencia) | **2**  Bastante (interferencia moderada; definitivamente interfiere en mi vida) | **3**  Mucha interferencia | **4**  Muchísimo (interferencia extrema; me limita e incapacita) |

**OCRD-D-E: ENGLISH VERSION**

**A. Thoughts/behaviors related to harm/responsibility and checking**

Have you ever had distressing thoughts, ideas or images about harm, possible catastrophic events, or ­­­responsibility? For example, about something bad happening to yourself or someone else, or about being responsible for or harming others. These kinds of thoughts often come in the­­ form of “what if…?” and can be accompanied by checking behaviors or other behaviors or mental acts performed to prevent or control possible harm. Such behaviors can include checking whether the door is locked, windows closed, or power outlets safe. Some people have to do mental rituals in response to the disturbing thoughts or images. You can feel forced to perform these behaviors over and over again. Seeking reassurance from others (e.g., parents, friends, partners) is also common; for example, by asking them to assure that you really did lock the door, closed the window, etc. It is common to avoid situations, objects or people that trigger the disturbing thoughts. For example, holding knives or standing close to people out of fear that you might push or harm them.

A1. Have you ever experienced these kinds of thoughts/behaviors (circle)?

**Yes No**

A2. Have you experienced these kinds of thoughts/behaviors during **THE LAST MONTH**?

(If not present during the last month, mark 0 for all questions)

| On average, how *frequently* do these thoughts/behaviors occur each day? | **0**  None | **1**  Mild (occasionally) | **2**  Moderate  (often) | **3**  Severe  (very often) | **4**  Extreme  (near constant) |
| --- | --- | --- | --- | --- | --- |
| How much *distress* do these thoughts/behaviors cause you? | **0**  None | **1**  Mild | **2**  Moderate | **3**  Severe | **4**  Extreme (disabling) |
| How hard is it for you to *control* these thoughts/behaviors? | **0**  Complete control | **1**  Much control | **2**  Moderate control | **3**  Little control | **4**  No control |
| How much do these thoughts/behaviors cause you to *avoid* doing anything, going anyplace, or being with anyone? | **0**  No avoidance | **1**  Occasional avoidance | **2**  Moderate avoidance | **3**  Frequent and extensive avoidance | **4**  Extreme avoidance (housebound) |
| How much do these thoughts/behaviors *interfere* with school, work or your social or family life? | **0**  None | **1**  Slight interference | **2**  Moderate (definitely interferes with functioning) | **3**  Much interference | **4**  Extreme interference (disabling) |

**B. Forbidden thoughts about sexuality, religion, or morality**

Have you ever had distressing thoughts that revolve around sexual, religious, or moral concerns? Religious thoughts can be about blasphemy or violation of religious rules or customs and can cause a need to confess or pray. Sexual thoughts can be about sexual orientation, sexual practices (e.g., sex with children or animals) or mental images of distressing sexual scenes or acts. Moral thoughts can be about a fear of doing something that you think is morally wrong or a feeling that you are morally reprehensible. These kinds of thoughts are often called taboo or forbidden thoughts. Many people feel the need to control their bodily reactions when they experience these kinds of thoughts and it is common to avoid situations, objects or people that trigger the thoughts.

B1. Have you ever experienced these kinds of thoughts (circle)?

**Yes No**

B2. Have you experienced these kinds of thoughts during **THE LAST MONTH**?

(If not present during the last month, mark 0 for all questions)

| On average, how *frequently* do these thoughts occur each day? | **0**  None | **1**  Mild (occasionally) | **2**  Moderate  (often) | **3**  Severe  (very often) | **4**  Extreme  (near constant) |
| --- | --- | --- | --- | --- | --- |
| How much *distress* do these thoughts cause you? | **0**  None | **1**  Mild | **2**  Moderate | **3**  Severe | **4**  Extreme (disabling) |
| How hard is it for you to *control* these thoughts? | **0**  Complete control | **1**  Much control | **2**  Moderate control | **3**  Little control | **4**  No control |
| How much do these thoughts cause you to *avoid* doing anything, going anyplace, or being with anyone? | **0**  No avoidance | **1**  Occasional avoidance | **2**  Moderate avoidance | **3**  Frequent and extensive avoidance | **4**  Extreme avoidance (housebound) |
| How much do these thoughts *interfere* with school, work or your social or family life? | **0**  None | **1**  Slight interference | **2**  Moderate (definitely interferes with functioning) | **3**  Much interference | **4**  Extreme interference (disabling) |

**C. Thoughts/behaviors related to symmetry, “not just right” feelings and repeating and ordering**

Have you ever had disturbing thoughts or feelings about a need for balance, symmetry, or evenness? These thoughts/feelings often relate to that objects, one’s own body or certain actions are experienced as "not just right” or "incomplete”. The thoughts and feelings can trigger you to repeat actions, order things, touch things in a certain way, or arrange things. Evening-out behaviors, counting and avoidance of situations, objects or people that trigger these distressing thoughts/behaviors/feelings are common.

C1. Have you ever experienced these kinds of thoughts/behaviors/feelings (circle)?

**Yes No**

C2. Have you experienced these kinds of thoughts/behaviors/feelings during **THE LAST MONTH**?

(If not present during the last month, mark 0 for all questions)

| On average, how *frequently* do these thoughts/behaviors/feelings occur each day? | **0**  None | **1**  Mild (occasionally) | **2**  Moderate  (often) | **3**  Severe  (very often) | **4**  Extreme  (near constant) |
| --- | --- | --- | --- | --- | --- |
| How much *distress* do these thoughts/behaviors/feelings cause you? | **0**  None | **1**  Mild | **2**  Moderate | **3**  Severe | **4**  Extreme (disabling) |
| How hard is it for you to *control* these thoughts/behaviors/feelings? | **0**  Complete control | **1**  Much control | **2**  Moderate control | **3**  Little control | **4**  No control |
| How much do these thoughts/behaviors/feelings cause you to *avoid* doing anything, going anyplace, or being with anyone? | **0**  No avoidance | **1**  Occasional avoidance | **2**  Moderate avoidance | **3**  Frequent and extensive avoidance | **4**  Extreme avoidance (housebound) |
| How much do these thoughts/behaviors/feelings *interfere* with school, work or your social or family life? | **0**  None | **1**  Slight interference | **2**  Moderate (definitely interferes with functioning) | **3**  Much interference | **4**  Extreme interference (disabling) |

**D. Thoughts/behaviors related to contamination, dirt, disgust, and cleaning behaviors**

Have you ever had distressing thoughts, ideas or images about contamination, germs and cleanliness? These thoughts can involve fears about being infected, getting poisoned, catching germs or spreading disease. Some people just feel disgusted. These thoughts can lead to a feeling that you need to wash your hands/other body parts, clean your things or separate “dirty” things from “clean” things. The thoughts can make it hard for you to touch things that others can touch (e.g., doorknobs). Further, they can make you spend a lot of time and energy in cleaning yourself or your things. Going to the bathroom can take a long time and can involve compulsive behaviors and avoidance of certain objects. It can also be hard to be near or handle chemicals or sticky substances. It is common to avoid situations, objects or people that trigger these thoughts/behaviors.

*When answering this question, think about your thoughts/behaviors in general, and not specifically those due to the current exceptional situation regarding the Covid-19 pandemic, where these thoughts and behaviors of hygiene and social distance are sanitary measures assumed by the whole society.*

D1. Have you ever experienced these kinds of thoughts/behaviors (circle)?

**Yes No**

D2. Have you experienced these kinds of thoughts/behaviors during **THE LAST MONTH**?

(If not present during the last month, mark 0 for all questions)

| On average, how *frequently* do these thoughts/behaviors occur each day? | **0**  None | **1**  Mild (occasionally) | **2**  Moderate  (often) | **3**  Severe  (very often) | **4**  Extreme  (near constant) |
| --- | --- | --- | --- | --- | --- |
| How much *distress* do these thoughts/behaviors cause you? | **0**  None | **1**  Mild | **2**  Moderate | **3**  Severe | **4**  Extreme (disabling) |
| How hard is it for you to *control* these thoughts/behaviors? | **0**  Complete control | **1**  Much control | **2**  Moderate control | **3**  Little control | **4**  No control |
| How much do these thoughts/behaviors cause you to *avoid* doing anything, going anyplace, or being with anyone? | **0**  No avoidance | **1**  Occasional avoidance | **2**  Moderate avoidance | **3**  Frequent and extensive avoidance | **4**  Extreme avoidance (housebound) |
| How much do these thoughts/behaviors *interfere* with school, work or your social or family life? | **0**  None | **1**  Slight interference | **2**  Moderate (definitely interferes with functioning) | **3**  Much interference | **4**  Extreme interference (disabling) |

**E. Concerns/behaviors related to flaws in physical appearance**

Have you ever had concerns or preoccupations about your physical appearance (perceived appearance flaws) or repetitive behaviors that you do in response to these concerns/preoccupations? The concerns/preoccupations are related to one or more perceived defects or flaws in your physical appearance that you yourself experience as very disturbing but that is not at all or only slightly observable to others. Repetitive behaviors (e.g., mirror checking, excessive grooming, skin picking, or asking for others’ opinions about one's appearance) or mental acts (e.g., comparing your appearance to others) in response to the appearance concerns are common. It is also common to avoid situations, objects, or people because of these concerns/behaviors and to try to hide the perceived flaws when other people are present.

The concerns/preoccupations should not be related solely to your body weight or shape or amount of body fat.

E1. Have you ever experienced these kinds of concerns/behaviors (circle)?

**Yes No**

E2. Have you experienced these kinds of concerns/behaviors during **THE LAST MONTH**?

(If not present during the last month, mark 0 for all questions)

| On average, how *frequently* do these concerns/behaviors occur each day? | **0**  None | **1**  Mild (occasionally) | **2**  Moderate  (often) | **3**  Severe  (very often) | **4**  Extreme  (near constant) |
| --- | --- | --- | --- | --- | --- |
| How much *distress* do these concerns/behaviors cause you? | **0**  None | **1**  Mild | **2**  Moderate | **3**  Severe | **4**  Extreme (disabling) |
| How hard is it for you to *control* these concerns/behaviors? | **0**  Complete control | **1**  Much control | **2**  Moderate control | **3**  Little control | **4**  No control |
| How much do these concerns/behaviors cause you to *avoid* doing anything, going anyplace, or being with anyone? | **0**  No avoidance | **1**  Occasional avoidance | **2**  Moderate avoidance | **3**  Frequent and extensive avoidance | **4**  Extreme avoidance (housebound) |
| How much do these concerns/behaviors *interfere* with school, work or your social or family life? | **0**  None | **1**  Slight interference | **2**  Moderate (definitely interferes with functioning) | **3**  Much interference | **4**  Extreme interference (disabling) |

**F. Difficulties/behaviors related to accumulation of items**

Have you ever had a persistent difficulty discarding or parting with possessions regardless of their actual value because you perceive a strong need to save them and/or because it would result in distress if you were to discard them? This difficulty of discarding items often results in the accumula­tion of a large number of possessions that congest and clutter active living areas to the ex­tent that their intended use is substantially compromised. Also, it is common to collect, buy or steal items that are not needed or for which there is no available space.

F1. Have you ever accumulated items as described above? (circle)?

**Yes No**

F2. Have you accumulated items as described above during **THE LAST MONTH**?

(If not present during the last month, mark 0 for all questions)

| To what extent do you have *difficulty discarding* (or recycling, selling, giving away) ordinary things that other people would get rid of? | **0**  None | **1**  Mild | **2**  Moderate | **3**  Severe | **4**  Extreme |
| --- | --- | --- | --- | --- | --- |
| How much *distress* do these difficulties/behaviors cause you? | **0**  None | **1**  Mild | **2**  Moderate | **3**  Severe | **4**  Extreme (disabling) |
| Because of the clutter or number of possessions, *how difficult* is it for you to use the rooms in your home? | **0**  Not at all difficult | **1**  Mild | **2**  Moderate | **3**  Severe | **4**  Extremely difficult |
| How much do these difficulties/behaviors cause you to *avoid* doing anything, going anyplace, or being with anyone? | **0**  No avoidance | **1**  Occasional avoidance | **2**  Moderate avoidance | **3**  Frequent and extensive avoidance | **4**  Extreme avoidance (housebound) |
| How much do these difficulties/behaviors *interfere* with school, work or your social or family life? | **0**  None | **1**  Slight interference | **2**  Moderate (definitely interferes with functioning) | **3**  Much interference | **4**  Extreme interference (disabling) |

**G. Urges/behaviors related to pulling your hair**

Have you ever had urges to pull out hair from your head or your body and/or have you ever pulled hair from your head/body (not with the purpose of harming yourself, and not out of esthetic reasons)? Hair pulling is characterized by recurrent pulling of hair that re­sult in hair loss and repeated attempts to decrease or stop pulling hair. These behaviors are not caused by specific distressing thoughts or preoccupations with appearance; however, they may be preceded or accompanied by various emotional states, such as feelings of anxiety, stress, or boredom. The behaviors may also be preceded by an increasing sense of tension and pulling hair may lead to gratification, pleasure, or a sense of relief. It is common to have vary­ing degrees of conscious awareness of the behavior while performing it. Sometimes you may be well aware of the behaviors (with preceding tension and subsequent relief), other times the behavior may be performed outside of awareness. It is common to avoid situations, objects, or people because these urges, behaviors, and hair loss.

G1. Have you ever experienced these kinds of urges/behaviors? (circle)

**Yes No**

G2. Have you experienced these kinds of urges/behaviors during **THE LAST MONTH**?

(If not present during the last month, mark 0 for all questions)

| On average, how *frequently* do these urges/behaviors occur each day? | **0**  None | **1**  Mild (occasionally) | **2**  Moderate  (often) | **3**  Severe  (very often) | **4**  Extreme  (near constant) |
| --- | --- | --- | --- | --- | --- |
| How much *distress* do these urges/behaviors cause you? | **0**  None | **1**  Mild | **2**  Moderate | **3**  Severe | **4**  Extreme (disabling) |
| How hard is it for you to *control* these urges/behaviors? | **0**  Complete control | **1**  Much control | **2**  Moderate control | **3**  Little control | **4**  No control |
| How much do these urges/behaviors cause you to *avoid* doing anything, going anyplace, or being with anyone? | **0**  No avoidance | **1**  Occasional avoidance | **2**  Moderate avoidance | **3**  Frequent and extensive avoidance | **4**  Extreme avoidance (housebound) |
| How much do these urges/behaviors *interfere* with school, work or your social or family life? | **0**  None | **1**  Slight interference | **2**  Moderate (definitely interferes with functioning) | **3**  Much interference | **4**  Extreme interference (disabling) |

**H. Urges/behaviors related to picking your skin**

Have you ever had urges to pick your skin or picked your skin (not with the purpose of harming yourself, and not because of esthetic reasons)? Skin-picking is characterized by recurrent picking of one's skin re­sulting in skin lesions and repeated attempts to decrease or stop the skin picking. These behaviors are not caused by specific distressing thoughts or preoccupations with appearance; however, they may be preceded or accompanied by various emotional states, such as feelings of anxiety, stress, or boredom. The behaviors may also be preceded by an increasing sense of tension and skin picking may lead to gratification, pleasure, or a sense of relief. It is common to have vary­ing degrees of conscious awareness of the behavior while performing it. Sometimes you may be well aware of the behaviors (with preceding tension and subsequent relief), other times the behavior may be performed outside of awareness. It is common to avoid situations, objects, or people because these urges, behaviors and skin lesions.

H1. Have you ever experienced these kinds of urges/behaviors? (circle)

**Yes No**

H2. Have you experienced these kinds of urges/behaviors during **THE LAST MONTH**?

(If not present during the last month, mark 0 for all questions)

| On average, how *frequently* do these urges/behaviors occur each day? | **0**  None | **1**  Mild (occasionally) | **2**  Moderate  (often) | **3**  Severe  (very often) | **4**  Extreme  (near constant) |
| --- | --- | --- | --- | --- | --- |
| How much *distress* do these urges/behaviors cause you? | **0**  None | **1**  Mild | **2**  Moderate | **3**  Severe | **4**  Extreme (disabling) |
| How hard is it for you to *control* these urges/behaviors? | **0**  Complete control | **1**  Much control | **2**  Moderate control | **3**  Little control | **4**  No control |
| How much do these urges/behaviors cause you to *avoid* doing anything, going anyplace, or being with anyone? | **0**  No avoidance | **1**  Occasional avoidance | **2**  Moderate avoidance | **3**  Frequent and extensive avoidance | **4**  Extreme avoidance (housebound) |
| How much do these urges/behaviors *interfere* with school, work or your social or family life? | **0**  None | **1**  Slight interference | **2**  Moderate (definitely interferes with functioning) | **3**  Much interference | **4**  Extreme interference (disabling) |
